# Immediate and Heterogeneous Response of the LiaFSR Two-Component System of *Bacillus subtilis* to the Peptide Antibiotic Bacitracin

**DOI:** 10.1371/journal.pone.0053457

**Published:** 2013-01-11

**Authors:** Sara Kesel, Andreas Mader, Carolin Höfler, Thorsten Mascher, Madeleine Leisner

**Affiliations:** 1 Center for NanoScience, Ludwig-Maximilians-University, Fakultät für Physik, Munich, Germany; 2 Department Biology I, Microbiology, Ludwig-Maximilians-University Munich, Planegg-Martinsried, Germany; Institut Pasteur, France

## Abstract

**Background:**

Two-component signal transduction systems are one means of bacteria to respond to external stimuli. The LiaFSR two-component system of *Bacillus subtilis* consists of a regular two-component system LiaRS comprising the core Histidine Kinase (HK) LiaS and the Response Regulator (RR) LiaR and additionally the accessory protein LiaF, which acts as a negative regulator of LiaRS-dependent signal transduction. The complete LiaFSR system was shown to respond to various peptide antibiotics interfering with cell wall biosynthesis, including bacitracin.

**Methodology and Principal Findings:**

Here we study the response of the LiaFSR system to various concentrations of the peptide antibiotic bacitracin. Using quantitative fluorescence microscopy, we performed a whole population study analyzed on the single cell level. We investigated switching from the non-induced ‘OFF’ state into the bacitracin-induced ‘ON’ state by monitoring gene expression of a fluorescent reporter from the RR-regulated *liaI* promoter. We found that switching into the ‘ON’ state occurred within less than 20 min in a well-defined switching window, independent of the bacitracin concentration. The switching rate and the basal expression rate decreased at low bacitracin concentrations, establishing clear heterogeneity 60 min after bacitracin induction. Finally, we performed time-lapse microscopy of single cells confirming the quantitative response as obtained in the whole population analysis for high bacitracin concentrations.

**Conclusion:**

The LiaFSR system exhibits an immediate, heterogeneous and graded response to the inducer bacitracin in the exponential growth phase.

## Introduction

Two-component systems (TCS) are a fundamental principle of bacterial signal transduction that enables cells to respond to environmental stimuli [1]–[3]. These phosphotransfer systems involve two conserved components, a histidine protein kinase (HK) and a response regulator protein (RR). Extracellular stimuli are sensed by the HK, leading to its autophosphorylation [4]. The phosphoryl group is then transferred from the HK to the RR. The RR, now in its ‘active’ form, elicits the specific response. Bacteria such as *Escherichia coli* or *Bacillus subtilis* posses about 30 HKs and RRs [5], [6], including well-known systems such as the EnvZ/OmpR TCS of the osmosensing pathway [7] or the HK CheA of the chemotaxis system phosphorylating two RRs, CheB and CheY [8]. In addition to functional characterization of TCS focusing on phosphorylation rates [9] accompanied by theoretical studies [10], [11], specificity and crosstalk of TCS is of great interest [12] and several methods for two-component research have been developed to accommodate such studies [13]. While some TCS mediate differential expression of the output genes by a graded response [7], others result in an all-or-nothing response [14]. The latter is only triggered after a particular stimulus concentration has been overcome. The response itself can thereby be homogeneous (the whole population behaves in the same way) or heterogeneous with parts of the population behaving differently than the others. Regardless of the observed output, regulation of both types of systems can involve a number of auxiliary protein components. Systems involving accessory proteins [15]–[17], often referred to as three-component systems, also include peptide antibiotic-sensing systems of Gram-positive bacteria [18], [19], [20].

**Figure 1:**
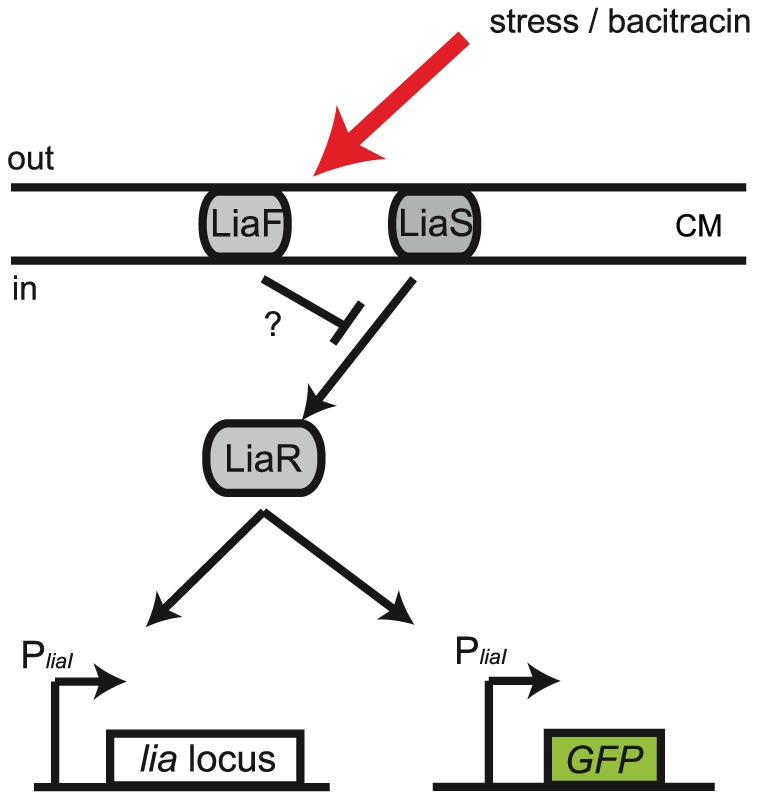
Core of the LiaFSR system. Arrows denote upregulation and T-shaped lines indicate inhibition. The LiaFSR system of *Bacillus subtilis* consists of the two-component signal transducing system LiaRS and the accessory membrane protein LiaF, a LiaRS-specific inhibitor. Stress represented e.g. by cell wall antibiotics such as bacitracin is sensed by LiaS/F and leads to expression of the *liaIH - liaGFSR* (“*lia* locus” in the Figure) locus mediated by LiaR. To study the response of the Lia system to external stressors, we report activity of P*_liaI_* using the fluorescent marker GFP expressed under the control of the *liaI* promoter, chromosomally inserted ectopically in addition to the native Lia system. CM indicates the cytoplasmic membrane.

One such system is the LiaFSR cell envelope stress response module of *Bacillus subtilis* [21], [22], which strongly responds to various peptide antibiotics such as bacitracin, nisin, vancomycin or daptomycin [23], but also to other less specific envelope perturbating conditions, such detergents or alkaline shock (summarized in [24] and [25]). The Lia system, is comprised of the LiaRS TCS, with the HK LiaS and the RR LiaR, and additionally the accessory protein LiaF (Figure 1). The latter is associated with all LiaRS-like TCS and acts as a negative regulator of LiaR-mediated gene regulation [21]. The mechanism by which LiaF interferes with LiaRS-dependent signal transduction is not yet understood. The genes of the LiaFSR system, together with a forth protein of unknown function, LiaG, are encoded in the *liaGFSR* operon, which is expressed from the constitutive *liaG* promoter (P*_liaG_*) in the absence of inducing conditions [21]. Activation of LiaR results in induction of the *liaI* promoter (P*_liaI_*) resulting in a strong upregulation of the *liaIH* operon, but also the complete *lia* locus (Figure 1) [21], [22]. The exact physiological role of LiaI and LiaH is not well understood, but the proteins seem to be involved in sensing and counteracting membrane damage [22]. In contrast to other cell wall antibiotic sensors of *B. subtilis*, such as the BceRS and PsdRS systems that directly sense peptide antibiotics and specifically mediate resistance against them [26], the Lia system seems to respond only indirectly to some quality of the damage caused by the diverse set of inducing conditions [27].

Here we focus on the activation of the P*_liaI_* by LiaR in response to the external stimulus bacitracin, which is the strongest and most robust inducer of LiaRS activity [23], [26]. As seen recently in other studies [28], [29], signal transduction of TCS can result in heterogeneous expression of genes regulated by these TCS. Heterogeneous gene expression in genetically identical cells can result in phenotypic different outcomes, a phenomenon also known as phenotypic heterogeneity [30]. Gene expression in itself is a stochastic or ‘noisy’ process [31]. Two different kinds of noise can be distinguished: intrinsic noise, due to noise in transcription or translation of the particular gene studied; or extrinsic noise as caused by fluctuations in the amount of other cellular components affecting gene expression [31]. Independent of the source of the noise, the arising heterogeneity can be manifested in broad gene expression distributions or by bifurcation into distinct subpopulations [32], as has been observed in *B. subtilis* in case of the transition state and stationary phase differentiation [32], [33].

For the LiaFSR system, averaged data obtained by whole population studies revealed that the response of the P*_liaI_* is dependent on the external antibiotic concentration [23]. However, a quantitative single cell analysis of the Lia response addressing heterogeneity in gene expression has not yet been performed. Using quantitative fluorescence microscopy [33], [34], we focused on a whole population study analyzed at the single cell level. We monitored gene expression from P*_liaI_* over time and found heterogeneity at low bacitracin concentrations. While expression levels from P*_liaI_* increased with the externally provided bacitracin amount, we found the immediate response of the LiaFSR system independent of the antibiotic concentration. We defined a switching threshold from the non-induced ‘OFF’ state to the bacitracin-induced ‘ON’ state. The number of cells in the ‘ON’ state, as well as the basal expression rate of the P*_liaI_* increased with bacitracin concentration. In addition, a well defined time window for switching into the ‘ON’ state was observed at all bacitracin concentrations.

## Results

### Gene expression increases at high bacitracin concentrations

In this study, we aimed at a deeper understanding of the response of the LiaFSR system to various concentrations of the peptide antibiotic bacitracin. We used the *B. subtilis* strain TMB 1172 [35], which carries a translational fusion of P*_liaI_* with the green fluorescent protein GFPmut1. This GFP reporter has been integrated chromosomally in addition to the naturally occurring genes under the control of P*_liaI_* and regulated by the RR LiaR (Figure 1). Therefore, we were able to study the response of the LiaFSR system by analyzing the expression of the GFP reporter, as it represents the expression of the LiaR regulated target genes. In particular, we studied the fluorescence development of the GFP reporter in dependence of bacitracin, a model component used to study cell envelope stress response modules of *Bacillus subtilis* [19], [36]. We chose the stable GFP variant, GFPmut1, shown to have a half-life of more than 24 h [37], [38], as we were only interested in the onset of gene expression. Thereby, we excluded possible variations in gene expression due to GFP decay.

**Figure 2:**
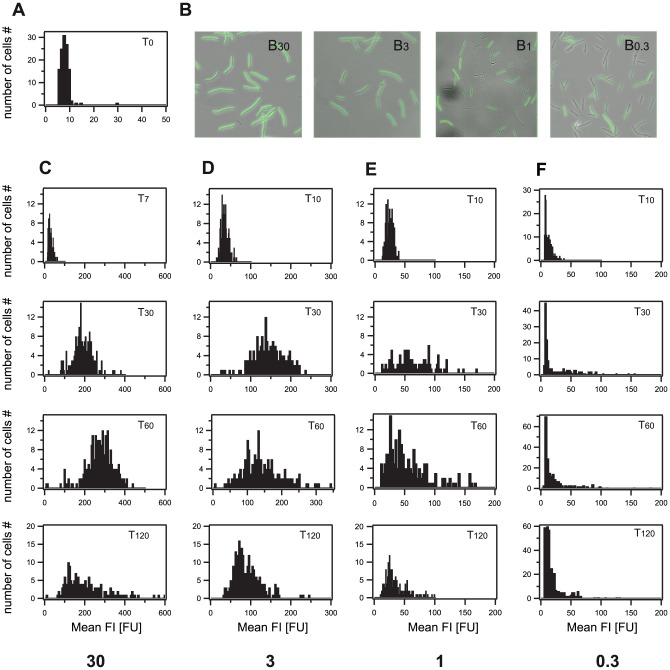
Expression profiles of the P*_liaI_* response in dependence of the bacitracin concentration. Addition of bacitracin induced GFP expression. At T_60_ all cells reached their maximum fluorescence intensities. While at high bacitracin concentrations all cells shifted to high fluorescence values, at low bacitracin concentrations (1 and 0.3 µg/ml) a fraction of cells did not express GFP. The observed decrease of fluorescence intensities after T_60_ is attributed to ongoing cell division. A) Autofluorescence (∼8 FU) of *Bacillus subtilis* cells recorded shortly before bacitracin addition at T_0_. B) Representative images of *B. subtilis* cells 60 min after bacitracin induction. Bacitracin concentration is given in the right upper corner of each image in µg/ml. C)–F) Histograms of GFP expression from the *liaI* promoter for different time points, at C) 30 µg/ml bacitracin (T_7_ = 7 min after bacitracin induction), D) 3 µg/ml bacitracin, E) 1 µg/ml bacitracin, and F) 0.3 µg/ml bacitracin.

Our cells were grown until mid-exponential phase before being induced with bacitracin to ensure that the recorded P*_liaI_* response was only due to external induction via bacitracin rather than intrinsic induction via the transition state regulator AbrB or the master regulator of sporulation Spo0A as present in the stationary phase [39]. Prior to bacitracin induction, we quantified the fluorescence intensity (FI) of non-induced cells representing the autofluorescence level (FI_auto_) and found it to be narrowly distributed with FI_auto_ 8±1 FU (Figure 2A). After bacitracin induction, we monitored the fluorescence development for two hours with five to seven minute intervals. At high bacitracin concentrations all cells shifted from the autofluorescence level to intermediate and finally high GFP expression levels. The maximal fluorescence intensities were reached at 60 min after bacitracin induction as shown in Figure 2B–F. While at 30 µg/ml bacitracin maximal fluorescence intensities of 272 FU on average were reached, FI_max_ decreased with lower bacitracin concentrations (Table 1). FI_max_ thereby represents the average FI of all cells at time point 60 min (see Materials and Methods). As seen in earlier publications [23], [36], we verified that even the highest bacitracin concentrations used had no negative effects on cell growth, thereby ruling out the risk of affecting gene expression (Figure S1). In addition, we performed control experiments using a promoter-less GFP mutant to ensure that the observed increase in fluorescence is due to bacitracin induction. As expected no GFP expression could be detected in the promoter-less mutant (data not shown).

**Table 1:** Quantitative Analysis of the LiaFSR response.

Bacitracin [µg/ml]	FI_max_ [FU]	f_ONmax_ [%]	P_fONmax_ [%/min]	t(P_fONmax_) [min]	FI_basalmax_ [FU]	Pa_max_ [FU/min]	t(Pa_max_) [min]
30	272±2	99±0.2	10.6±1.1	11.3±1.3	NA	NA	NA
3	139±4	100±0	19.5±13.8	10.6±1.9	NA	NA	NA
1	44±4	78±2.4	9.7±2.9	14.2±1.1	21.6±0.7	2.3±0.4	8.0±0.35
0.3	26±2	26±2	3.9±2.6	14.3±1.5	11.9±0.4	0.3±0. 3	6.2±1.3
0.1	8±1	2.3±0.1	NA	NA	NA	NA	NA

FI_max_ = average maximal fluorescence intensity at T_60_, f_ONmax_ = maximal fraction of cells in the ‘ON’ state, p_fONmax_ = maximal switching rate, t_(pfONmax)_ = time point of maximal switching, FI_basalmax_ = average maximal basal fluorescence intensity, Pa_max_ = maximal expression rate, t_(Pamax)_ = time point of maximal expression rate.

The general response of P*_liaI_* was similar for all bacitracin concentrations (Figure 2C–F). First, the whole cell population responded within less than 10 min as at T_10_ a clear shift to higher fluorescence values was observable. Only at very low bacitracin concentrations (0.1 µg/ml) hardly any fluorescence could be detected within the 120 min observation period, as cells stayed at FI_auto_ = 8±1 FU (Figure S2). Second, FI_max_ was reached within 60 min. Third, after 60 min fluorescence levels decreased again probably due to ongoing cell division. Taken together our data demonstrate that the LiaFSR system exhibits a graded and fast response to the external stimulus bacitracin: The FI_max_ as obtained after 60 min of induction increased with the stimulus concentration. In addition, cells started expression of the fluorescent protein even at low inducer concentrations within less than 10 min, in contrast to other systems such as e.g. the arabinose utilization system where for low inducer concentrations cells responded only 20 min after induction [40].

### Heterogeneity in gene expression is established at low bacitracin concentrations

**Figure 3:**
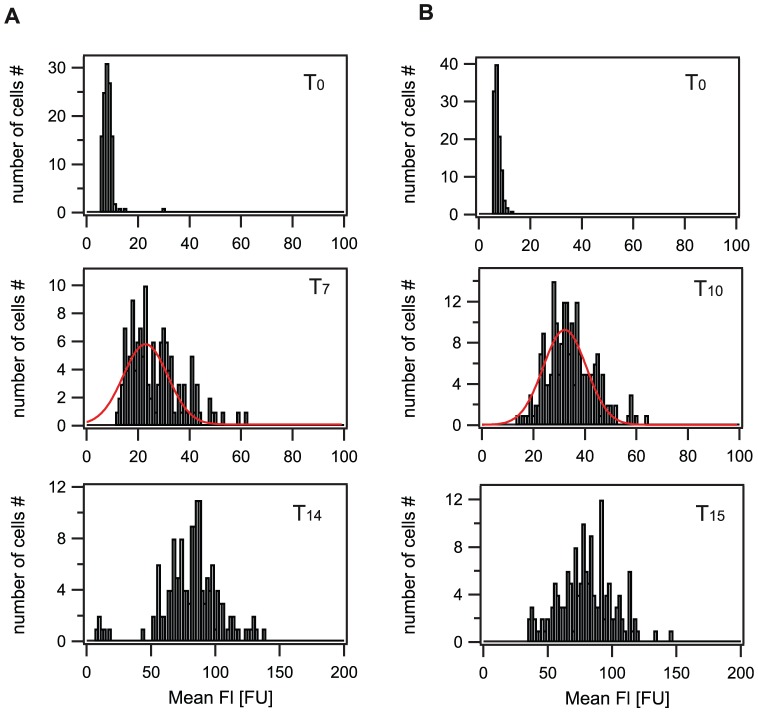
Definition of the switching threshold. Histograms of GFP fluorescence intensity at various time points. A) 30 µg/ml bacitracin, B) 3 µg/ml bacitracin. T_0_: time point of bacitracin induction representing the autofluorescence with ∼8 FU. T_7_ and T_10_: Time points 7 and 10 min after bacitracin induction representing the phase at which cells are switching into the ‘ON’ state. At T_14_ and T_15_ (14 and 15 min after bacitracin induction) all cells have switched and the fluorescence distribution is clearly shifted towards higher fluorescence values. T_7_ and T_10_ therefore represent intermediate switching states and have therefore been used to determine the switching threshold as described in the Materials and Methods section. Red line: Gaussian fit. For details on the fit parameters see Table S1.

**Figure 4:**
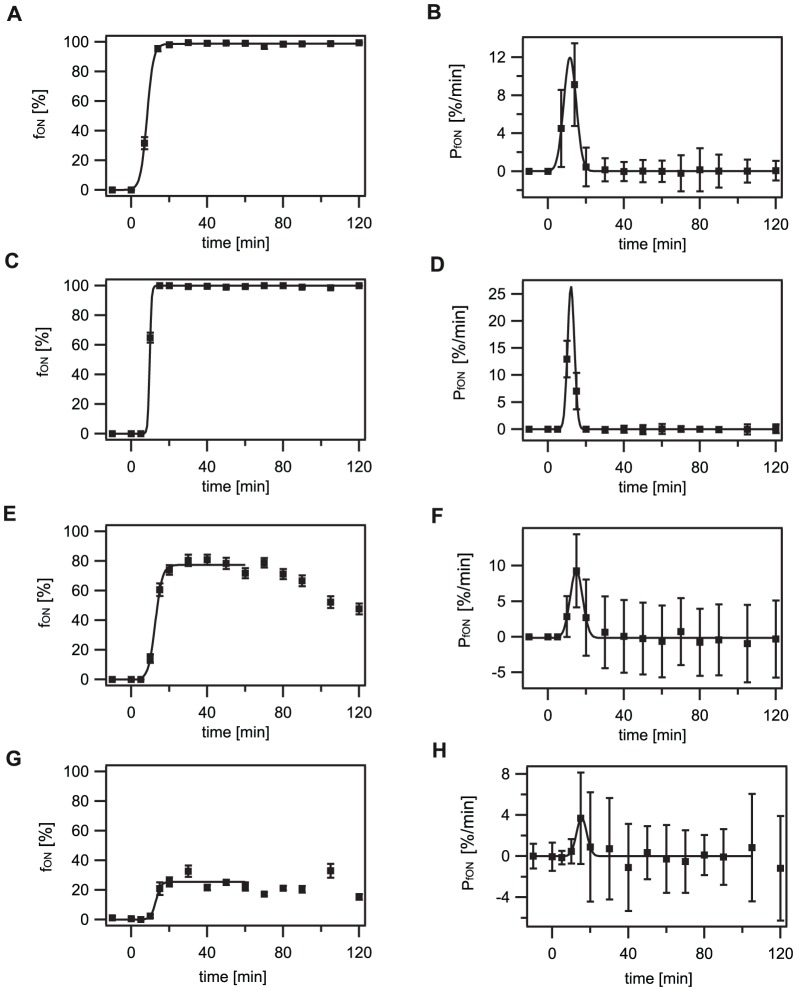
Fraction of cells in the ‘ON’ state as a function of time (f_ON_(T)) and switching rate (P_fON_). For definition of the switching threshold see description in the Materials and Method section. The fraction of cells in the ‘ON’ state (f_ON_) increased with time, finally saturating at its maximal level. The maximal fraction of cells in the ‘ON’ state (f_ONmax_) decreased with the bacitracin concentration. Similarly, the maximal switching rate (P_fONmax_) decreased at low bacitracin concentrations (e.g. 0.3 µg/ml). A, C, E, G) Fraction of cells in the ‘ON’ state as a function of time (f_ON_). Solid line: best fit to a sigmoid function as previously described in [33] (Table S2). B, D, F, H) Switching rate (P_fON_). The switching rate was determined as the first derivative with respect to time of the fraction of cells in the ‘ON’ state. Solid line: best fit to a Gaussian function (Table S3). A and B: 30 µg/ml bacitracin; C and D: 3 µg/ml bacitracin; E and F: 1 µg/ml bacitracin, G and H: 0.3 µg/ml bacitracin.

As we had observed that FI_max_ decreased with lower bacitracin concentrations, the question arose whether this was due to general lower fluorescence intensities in all cells at T_60_ or due to a heterogeneous GFP expression in the population at low inducer concentrations, with only a fraction of cells expressing GFP at high levels. While for high bacitracin concentrations (30 and 3 µg/ml) all cells switched from FI_auto_ to FI_max_ by 60 min post-induction, this could not be observed at low bacitracin concentrations (1 and 0.3 µg/ml). Here, parts of the population were not induced by bacitracin, as indicated by fluorescence levels in the range of the autofluorescence. Therefore, a clear heterogeneity in gene expression levels was present at 60 min after bacitracin induction at low antibiotic concentrations (Figure 2B). Interestingly, no bimodality was observed at any time point for low bacitracin concentrations, as FI levels of cells expressing GFP ranged continuously from FI_auto_ to high FI values, making it difficult to separate the non-induced cells from cells with induced GFP expression corresponding to higher GFP levels. Therefore, we defined the switching threshold from the non-induced ‘OFF’ state to the induced ‘ON’ state in the following way: At high bacitracin induction all cells switched into the induced “ON” state. Although FI_max_ was not reached until T_60_, all cells had clearly shifted away from the autofluoresce level FI_auto_ at T_7_ (30 µg/ml bacitracin) and T_10_ (3 µg/ml bacitracin). We used these intermediate states as seen in experiments with high inducer concentrations (30 and 3 µg/ml bacitracin) to determine the switching threshold by applying a Gaussian fit to the histograms shown in Figure 3 (see Material and Methods, Table S1). This resulted in a switching threshold of 30 FU: cells showing expression levels above 30 FU ( = three-fold above background) were considered as being in the ‘ON’ state. This threshold definition best reflected the observed fluorescence expression distributions (Figure 2C–F). Subsequently, we determined the fraction of cells in the ‘ON’ state as a function of time (f_ON_(T)) (see Materials and Methods), which was well described by a sigmoid function (Figure 4 left, Table 1, Table S2). Around 20 min after bacitracin induction, the fraction of cells in the ‘ON’ state saturated at f_ONmax_, ranging from 100% for high bacitracin concentrations to 2.3% for very low (0.1 µg/ml) antibiotic concentrations (Figure 4 left, Table 1, Figure S2). After these 20 min no further increase of the fraction of cells in the ‘ON’ state could be detected. The observed decrease of fluorescence intensities, and with it the fraction of cells in the ‘ON’ state, seen for low bacitracin concentrations (1 and 0.3 µg/ml), can be attributed to ongoing cell division. Our data show that the number of cells switching into the ‘ON’ state is dependent on the external antibiotic concentration and reaches a saturating level at 3 µg/ml bacitracin. Above this concentration all cells enter the ‘ON’ state.

### Switching into the ‘ON’ state occurs within 20 min

We next investigated the time needed by the whole population to switch into the ‘ON’ state by analyzing the switching rate (Materials and Methods). We determined the switching rate (P_fON_) as the first derivative of the fraction of cells in the ‘ON’ state with respect to time (Figure 4, right), which was well described by a Gaussian function (Material and Methods, Table S3). Maximal switching into the ‘ON’ state was observed at about 11 min for 3 and 30 µg/ml bacitracin and about 14 min for 1 and 0.3 µg/ml bacitracin. One possible explanation for this observation is heterogeneous timing [36]. Here, the time point of switching for individual cells is distributed over a longer time period. As the fraction of cells in the ‘ON’ state saturated 20 min after bacitracin induction, even for low bacitracin concentrations, and no further increase of the fraction of cells in the “ON” state could be observed thereafter, we find this explanation unlikely. Instead, we assume that cells still responding at low antibiotic concentrations need more time to do so (Figure 4 left, Table S4). The maximal switching rate (P_fONmax_) was about 10 to 20%/min for high bacitracin concentrations (Table 1, Table S5), and was significantly reduced at 0.3 µg/ml bacitracin with about 4%/min. Therefore, the small number of cells entering the ‘ON’ state at this bacitracin concentration can be ascribed to the reduced switching rate.

Independent on the bacitracin concentration added, switching into the ‘ON’ state started approximately five minutes after bacitracin induction, ending 20 min later. This indicates the presence of a well-defined switching window of about 20 min in which cells can enter the ‘ON’ state. As soon as bacitracin, or any damage caused by it, is sensed by the LiaFSR system, cells start to switch into the ‘ON’ state. The shut-down of the LiaFSR response can be understand in the context of the complete bacitracin stress response network that the Lia system is embedded in: several TCS are present in *B. subtilis* [19] that sense the antibiotic bacitracin leading to the activation of bacitracin detoxification systems that remove the antibiotic from its site of action [19], [32]. This in turn lowers the inducing stress that is sensed by the LiaFSR system, resulting in the observed ‘switch-off’ at about 20 min. Although, the fraction of cells in the ‘ON’ state does not increase any further 20 min after bacitracin induction, an increase in fluorescence intensities can be observed until T_60_. We attribute this to the stability of the GFP-mRNA: as long as GFP-mRNA is present, translation can occur, resulting in the obtained increase in fluorescence intensity.

### Basal expression rate of P*_liaI_* is dependent on bacitracin concentration

We observed that the maximal switching rate P_fONmax_ was reduced at 0.3 µg/ml bacitracin as compared to higher bacitracin concentrations and was reached at later time points. This raised the question whether the smaller switching rate at low bacitracin concentrations was due to a reduced P*_liaI_* promoter activity. We addressed this question by analyzing the basal expression rate (Pa). As GFPmut1 and LiaI represent two different proteins, it is possible that GFPmut1 and LiaI have different proteolysis rates. Therefore, the concentration of GFPmut1 controlled by P*_liaI_* is not necessarily a direct measure for the concentration of LiaI. However, the expression rates, i.e. the production rate of LiaI and GFPmut1, are expected to be similar, as the complete native P*_liaI_* including all native signals for LiaI expression is present.

**Figure 5:**
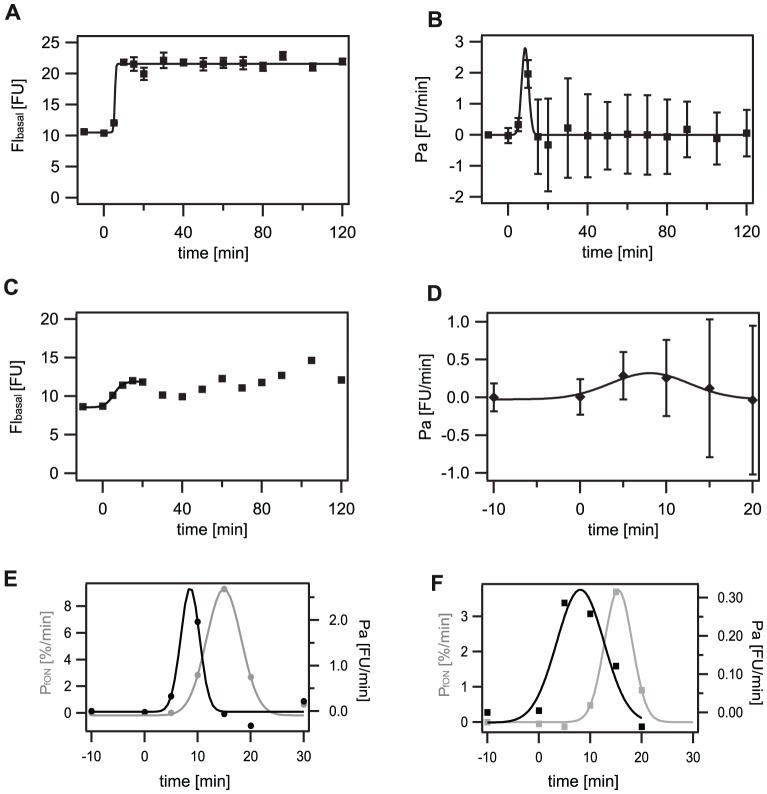
Basal expression rate (Pa) of P*_liaI_* at 1 and 0.3 µg/ml bacitracin. The average fluorescence intensities (FI_basal_) of cells in the ‘OFF’ state increased with time, saturating shortly thereafter. This enabled us to determine the basal expression rate (Pa) as described in the Material and Methods section. The maximal basal fluorescence intensity decreased with lower bacitracin concentrations. Similarly, the basal expression rate was significantly reduced in experiments with 0.3 µg/ml bacitracin as compared to 1 µg/ml bacitracin. A) and C) Fluorescence development of cells being in the ‘OFF’ state (FI_basal_). Solid line: best fit to a sigmoid function (Table S6). B) and D) Expression rate of P*_liaI_* as the first derivative of fluorescence development given in A) and C). Solid line: best fit to a Gaussian function (Table S7). A) and B): 1 µg/ml bacitracin, C) and D) 0.3 µg/ml bacitracin. E) and F) comparison of switching rate P_fON_ (grey) and basal expression rate Pa (black). E) 1 µg/ml bacitracin. F) 0.3 µg/ml bacitracin.

As a first step, we selected the cells that had not switched into the ‘ON’ state, as present in experiments with 1 and 0.3 µg/ml bacitracin. The average basal fluorescence value of cells that had not switched (FI_basal_) shifted to higher values with time, saturating at the maximal basal fluorescence value FI_basalmax_. This increase of fluorescence values of not-induced cells could be well described by a sigmoid fit function FI(T) (Table S6), similar to the fraction of cells in the ‘ON’ state. However, FI(T) was shifted towards earlier times as compared with f_ON_(T), indicating that the basal expression rate Pa had a maximum and that the maximum expression rate was shifted to earlier times as compared with the maximum switching rate P_fON_. The maximal fluorescence values of not-induced cells as obtained at 20 min after bacitracin induction showed significantly higher values as compared to the autofluorescence (Figure 5 A,C), with about 22 and 12 FU for 1 and 0.3 µg/ml bacitracin, respectively (Table 1).

We determined the basal expression rate Pa as the first derivative with respect to time of the mean grey value of those cells that had not entered the ‘ON’ state (Figure 5 B and D), which was well described by a Gaussian function (Table S7). The maximum basal expression rate, Pa_max_ (Material and Methods), at 1 µg/ml was 2.3±0.4 FU/min exceeding the value of 0.3±0.3 FU/min at 0.3 µg/ml bacitracin by a factor of eight (Table S8). This indicated that the graded response of the LiaFSR system was merely due to a decreased basal expression rate at low bacitracin concentrations. As the maximal basal expression rate was reached at about 7 min at 1 and 0.3 µg/ml bacitracin as compared to the maximal switching rate at about 14 min (Table 1, Table S9), switching into the ‘ON’ state can be attributed to the increase of the basal expression rate at these bacitracin concentrations. As the basal expression rate is reduced again to zero approximately 15–20 min after bacitracin induction, the duration of the switching window is well defined. The time delay between Pa_max_ and P_fONmax_ of about 6 to 8 min (Figure 5 E, F) is in the range of the maturation time of the used fluorescent protein GFPmut1 with 8 min (Figure S3, Table S10), demonstrating the immediate response of the LiaFSR system to the antibiotic bacitracin.

### Switching initiation is similar for individual cells

**Figure 6:**
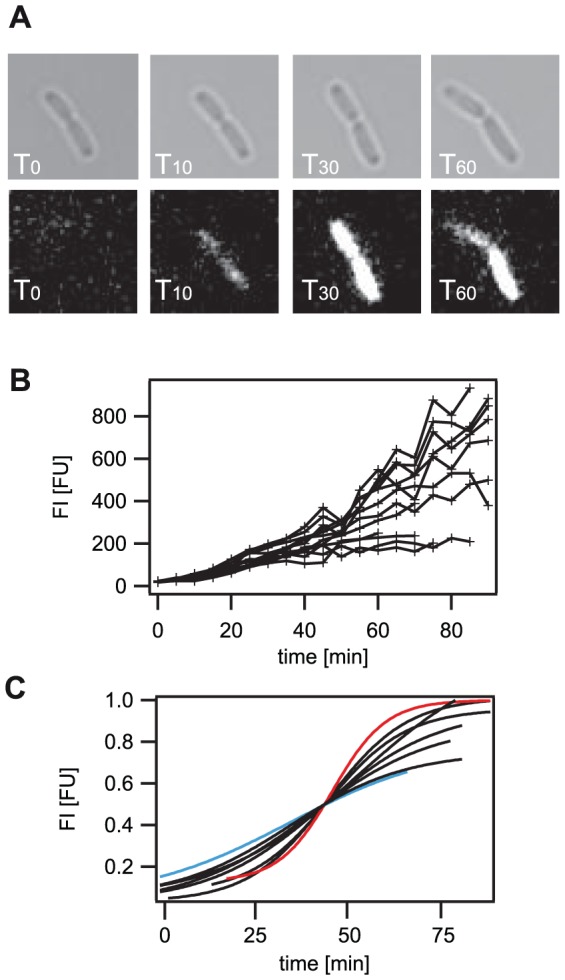
Switching characteristics of single cells at 30 µg/ml bacitracin. Fluorescence development of single cells over time at 30 µg/ml bacitracin was comparable to the data obtained by single cell analysis of the above described population study: All cells switched into the induced ‘ON’ state, exceeding the threshold fluorescence intensity within 15 min. In contrast to the whole population study the maximal fluorescence intensity was reached only after 80 min. A) Fluorescence development of one individual cell is shown. Top: bright field images at different time points. Bottom: fluorescence images at different time points. B) Fluorescence development of 13 individual cells is shown. C) Sigmoidal fits have been applied to eight fluorescence intensity traces in Figure 6B. The fluorescence intensity was normalized to the maximum fluorescence intensity and the time axis was shifted to T_45_, where cells had half-maximum fluorescence intensity. Blue and red line: two individual fluorescence traces representing cells with the slowest and highest individual switching rates in this cell batch.

So far, we have quantitatively analyzed the P*_liaI_* response of the whole bacterial population grown in stirred liquid cultures as given by the averaged values of the single cells. In order to study the switching behavior of individual cells we developed a new protocol for fluorescent time-lapse microscopy of exponentially growing *B. subtilis* cells. Bacteria were fixed via attachment to microfluidic chambers coated with a specific silane (Materials and Methods) and flushed with fresh medium including the antibiotic bacitracin. As bleaching of the GFPmut1 molecules in single cells was significant, we corrected the obtained fluorescent values as described in the Materials and Methods section. Since the GFP expression levels for low bacitracin concentrations were in the range of the bleaching, we were only able to monitor the switching behavior of individual cells over time at 30 µg/ml bacitracin. Analyzing bleach-corrected fluorescence values (Material and Methods), we observed that cells started switching at about five minutes after bacitracin induction and all cells had switched into the ‘ON’ state within 15 min, as seen in experiments performed in liquid cultures. As expected, individual cells reached fluorescence values at 60 min post-induction between 200 and 600 FU (Figure 6). But in contrast to the experiments of whole populations described above, FI values increased until 80 min (200–800 FU) indicating that cell division was reduced for cells grown directly on the microscopic slide rather than in flask cultures. Nevertheless, the same overall switching behavior could be observed for individual cells growing in the microfluidic chamber as compared to cells grown in liquid culture, demonstrating the suitability of this approach. In a next step we compared the individual switching curves by applying a sigmoid function to the fluorescence development of single cells over time. This study revealed that cells initiated switching into the ‘ON’ state within the same time frame, but the individual switching curves showed a high variation with individual switching rates ranging from 6–15 FU/min (Figure 6). In accordance with our findings of whole population studies, our single cell data obtained by time-lapse microscopy demonstrate the fast response of the LiaFSR system to bacitracin.

## Discussion

In this report, we quantitatively investigated the response of the LiaFSR system to an external signal, the peptide-antibiotic bacitracin, by performing a population study analyzed on the single cell level. Quantitative fluorescence microscopy (QFM) as described in this study, has been used previously to analyze switching of *Bacillus subtilis* into the competent state [33]. In this particular case it was shown that the sensitivity of this approach is high enough to detect an increase of promoter activity by a factor of two. This result was confirmed independently, using fluorescence in situ hybridization (FISH), demonstrating the usability of quantitative fluorescence microscopy [41]. Another quantitative method to analyze single cells is flow cytometry. We performed flow cytometry experiments in order to study the LiaFSR response to various bacitracin concentrations (Figure S4), confirming our results obtained by QFM. Fluorescence values of single cells obtained by flow cytometry for low bacitracin concentrations were difficult to separate from the buffer background even after applying gating procedures. Therefore, we chose to focus on quantitative fluorescence microcopy to analyze our data in order to obtain the complete information of the LiaFSR response for high and low bacitracin concentrations.

We observed an immediate response of the system with cells switching in the bacitracin-induced ‘ON’ state within 20 min, irrespective of the externally provided bacitracin concentration. The switching rate shows its maximum approximately 7 min after the maximum of the basal expression rate. Importantly, this response time is in the range of the maturation time of the green fluorescent reporter with 8 min [42], indicating an almost instant burst of LiaR-dependent transcription initiation at P*_liaI_*. This is in contrast to other studies, in which maximum RR-regulated mRNA concentrations [1] or the concentration of promoter-bound RR [10] could be detected only within 20–30 min after exposure to the externally provided signal. Functional characterization of all two-component signal transduction systems in *E. coli* revealed a wide span in auto-phosphorylation rates of the HK ranging from about 2 min to 10 min. Phospo-transfer to RRs by phosphorylated cognate HKs took place within less than ½ min [9]. As maximal switching into the ‘ON’ state of the LiaFSR system can be observed within 15 min after bacitracin addition, even at the lowest bacitracin concentration, this demonstrates that no further regulatory elements are involved in the bacitracin-dependent LiaFSR response. This is in line with our finding that the basal expression rate of the *liaI* promoter is dependent on the bacitracin concentration, indicating that the LiaR concentration is directly affecting gene expression from P*_liaI_*. Recently, it was found that even at very high bacitracin concentrations (50 µg/ml) only about 20 molecules of LiaR are present within a single cell [43], while in the absence of bacitracin LiaR was not detectable. The amount of available LiaR controlling expression from P*_liaI_* is therefore dependent on the bacitracin concentration. The low number of LiaR molecules can explain the observed variations in gene expression, in particular the heterogeneity present at low bacitracin concentrations, as cell-to-cell differences (noise [31]) in the exact number of LiaR directly affect gene expression from P*_liaI_*.

Performing a population study analyzed at the single cell level, in combination with time-lapse microscopy, we quantitatively analyzed the response of the LiaFSR two-component system to bacitracin. As described above, the LiaFSR system responds within less than 15 min to the external stimulus. Cell-to-cell differences are present at all bacitracin concentrations and decrease at low bacitracin levels. The maximum switching rate as well as basal expression rate depends on the bacitracin concentration, reflecting the graded response of the LiaFSR system. For a stress sensor system, this kind of response is reasonable. Changing environmental conditions, including the presence of stressors, require fast stress sensing systems such as the LiaFSR system, that are shut-off as soon as the stressor is no longer present. Taken together, our data demonstrate that the LiaFSR system exhibits an immediate, heterogeneous and graded response to the peptide antibiotic bacitracin in the exponential growth phase.

## Materials and Methods

### Growth conditions

*Bacillus subtilis* strain TMB 1172 [35] carries a translational fusion of P*_liaI_* with the green fluorescent reporter protein GFPmut1. TMB 1172 was grown in LB medium at 37°C, shaken at 300 rpm. Overnight cultures were diluted to OD_600_ of 0.1. Cells were grown to mid-logarithmic phase, then were again diluted to OD_600_ of 0.1 into fresh medium and grown for additional 30 min to ensure optimal growth conditions before induction with the peptide-antibiotic bacitracin (Sigma) at T_0_ = 30 min and applying the cells to the microscopic slides. This way any cross-over from intrinsic stationary phase induction [35] could be avoided. Experiments for each bacitracin concentration were performed in triplicates on three different days. For each time point a minimum of 100 cells was analyzed. The bacitracin concentrations used in this study are far below the minimal inhibitory concentration (MIC) [23], [36] and have been shown to have no effect on growth (Figure S1).

### Construction of promoter-less-gfp mutant strain

The promoter less vector pGFPamy [44] was transformed into *B. subtilis* as a negative control. The vector carries a chloramphenicol resistance cassette for selection in *B. subtilis*, and integrates into the *amyE* locus by double crossing-over, resulting in a stable integration of the promoter-less-gfp fusion. The plasmid was linearized with PstI and used to transform *B. subtilis* 168 with chloramphenicol selection (5 µg/ml). Successful integration into the *amyE* locus was confirmed by starch test.

### Flow cytometry

For flow cytometry experiments, the cultures were grown as described above. Samples were taken every 10 min for 120 min and diluted 1∶100 in PBS (phosphate buffered saline). The experiments were performed using a Partec CyFlow Space instrument and the software FlowMax. GFP was excited with a laser at 488 nm and its emission measured at 518 nm. The analysis of the cells was done at a flow-rate of 2 µl/s. In between measurements, the instrument was rinsed with PBS to eliminate cross-contamination. In addition to the different concentrations of bacitracin, not induced samples and PBS alone were analyzed for control purposes. To discriminate dead from healthy cells, appropriate gating procedures have been applied. 50000 cells lying in the appropriate gate have been analyzed for each time point.

### Fluorescence Microscopy

Cells were sampled throughout growth as indicated in the main text. For image acquisition of the whole cell population, cells were permitted to attach to microscopic slides (eight-well IBIDI chamber, uncoated) and covered with 1% Agarose-patches.

For time-series of single cells, cells were allowed to attach to microfluidic chambers coated with 100% 1-[3-(Trimethoxysilyl)propyl]urea (Sigma). Cells were induced already attached to the microfluidic channels and washed with fresh medium in the presence of bacitracin at a flow-rate of 0.3 ml/h.

Image acquisition was done using a Zeiss Axiovert 200 M microscope equipped with an Andor Digital Camera and a Zeiss EC Plan-Neofluar 100×/1.3 Oil immersion objective. Andor software was used for image acquisition. The stability of the absolute fluorescence values was verified using a microscope image intensity calibration kit (Invitrogen, FokalCheck™ fluorescence microscope test slide #3). Microspheres showed a deviation of mean grey value of less than 1% under the experimental conditions used for detection of GFP fluorescence. Homogeneity of illumination was tested using fluorescent slides and the maximum deviation was less than 5%.

### Image Analysis

Images were processed using ImageJ software. Image background was corrected using a rolling ball algorithm with a radius of 50. An intensity threshold tool was used to delimit the boundaries of the cells in the bright field image. The boundaries of the cells were obtained with the wand tool of the ImageJ software and transferred to the fluorescence image using the ROI manager of ImageJ. Only cells that were fully lying within the bright field image and were not in the process of cell division were considered. Furthermore, dead cells as observable by different contrast in the bright field image as compared to healthy cells were excluded from the single cell analysis. The remaining single cells were than analyzed with respect to their mean grey value. Data preparation was performed using the Software IGOR PRO 4.06 and Adobe Illustrator CS4.

### Definitions and calculation methods

*FI_auto_:* average autofluorescence/fluorescence intensity of cells not induced by bacitracin, given as fluorescence units [FU] as obtained by the mean grey value. The average autofluorescence level of cells prior bacitracin induction was FI_auto_ = 8±1 FU (Figure 2A).

*FI_max_:* average maximal GFP expression/fluorescence intensity as observed at T_60_. Upon induction with bacitracin cells expressed the GFP reporter. The resulting fluorescence intensities were obtained as the mean grey value of each single cell. The error of FI_max_ is given as the standard error.

*Switching threshold:* The switching threshold separates cells being in the ‘OFF’ state (no/basal expression) from cells being in the ‘ON’ state (induced GFP-expression). We used the intermediate states seen in experiments with high inducer concentrations (30 and 3 µg/ml bacitracin) to determine the switching threshold. A Gaussian fit was applied to the histograms shown in Figure 3 (Table S1). The values of the center of these distributions, in addition to the average fluorescence values of all cells at this time point, were averaged. The resulting value of 30 FU was then defined to be the switching threshold: any cell with fluorescent value above 30 FU (mean grey value) was considered as being in the ‘ON’ state.

*f_ON_:* fraction of cells in the ‘ON’ state. We determined the fraction of cells in the ‘ON’ state as a function of time using the switching threshold. The fraction of cells in the ‘ON’ state was well defined by a sigmoid function with f_ON_(T) = f_base_+f_max_/1+exp(*k*(T_half_−T)). The fit parameter of this function can be found in Table S2. The maximal fraction of cells in the ‘ON’ state (f_ONmax_) was determined using this fit function (Table S2). The error of the fraction of cells in the ‘ON’ state has been calculated according to: square root of (p(1−p)/(n−1)).

*P_fON_:* average switching rate of cells switching into the ‘ON’ state. The switching rate was determined as the first derivative of the fraction of cells in the ‘ON’ state with respect to time. To reduce the error, the maximal switching rate (P_fONmax_) was determined using two different calculation methods: a) P_fonmax_ = maximum of the 1^st^ derivative of the exact data points of f_ON_. b) by obtaining A of the Gaussian fit applied to the data Figure 4 right according to P_fON_ = y_0_+Aexp (−((x−x_0_)/width)^2^) (Table S5). The high error for data determined at 3 µg/ml bacitracin is attributed to the steep increase of the fraction of cells in the ‘ON’ state leading to a high fitting error. Additional data points in order to reduce the error could not be attained, as cells stored on ice for later image acquisition tended to lyse at bacitracin concentrations >1 µg/ml. Therefore image acquisition was performed immediately after sampling of the cells. The exact results of both calculation methods as well as the average values are given in Table S5. The error of the switching rate was calculated according to: Error P_fON_ at time point t_2_ = square root of ((error at (t_2_))^2^+(error at (t_1_))^2^), with t_1_ and t_2_ the time points of the derivated time interval. The individual errors here are the errors of f_ON_ as described above. Please note that error propagation has to be taken into account when deriving data points, leading to the high errors in Figure 4, F and H.

*t(P_fONmax_):* Time point of maximal switching rate. To reduce the error the time point of the maximal switching rate has been determined in three different ways: a) T_half_ of the sigmoidal fit applied to Figure 4 left according to f_ON_(T) = f_base_+f_max_/1+exp(k(T_half_−T)). b) Time point of P_fONmax_ = maximum of the 1^st^ derivative of the exact data points of f_ON_. c) by obtaining x_0_ of the Gaussian fit applied to the data in Figure 4 right according to P_fON_(T) = y_0_+A exp (−((x−x_0_)/width)^2^) (Table S4).

*FI_basal_:* average basal fluorescence intensity of cells in the ‘OFF’ state. The error is given as the standard error.

*FI_basalmax_:* maximal average basal fluorescence intensity as obtained by applying a sigmoidal fit function FI (T) = f_base_+f_max_/1+exp(*k*(T_half_−T)), with f_base_ baseline, f_max_ maximum basal fluorescence intensity, T_half_ half time and *k* rate (Table S6).

*Pa:* average basal expression rate of cells in the non-induced ‘OFF’ state. We determined the Pa as the first derivative with respect to time of the mean grey value of those cells that had not entered the ‘ON’ state. To reduce the error the maximal basal expression rate (Pa_max_) has been determined in two different ways: a) Pa = maximum of the 1^st^ derivative of the exact data points of FI_basal_. b) by obtaining x_0_ of the Gaussian fit applied to Figure 5 B, D according to Pa(T) = y_0_+Aexp (−((x−x_0_)/width)^2^) (Table S8). The error of the basal expression rate was calculated according to: Error Pa at time point t_2_ = square root of ((error at (t_2_))^2^+(error at (t_1_))^2^), with t_1_ and t_2_ the time points of the derivated time interval. The individual errors here are the errors of FI_basal_ as described above. Please note that error propagation has to be taken into account when deriving data points, leading to the high errors in Figure 5 B and D.

*t(Pa_max_):* The time point of the maximal basal expression rate has been determined in three different ways: a) T_half_ of the sigmoidal fit applied to Figure 5 A, C according to FI(T) = f_base_+f_max_/1+exp(k(T_half_−T)) (Table S9). b) Pa_max_ = maximum of the 1^st^ derivative of the exact data points of FI_basal_. c) by obtaining x_0_ of the Gaussian fit applied to Figure 5 right according to Pa(T) = y_0_+Aexp (−((x−x_0_)/width)^2^) (Table S9).

A summery of all data described here, as well as the average data obtained from the different calculation methods for t(P_fONmax_), t(Pa_max_), P_fONmax_ and Pa_max_ can be found in Table 1. The obtained data for each calculation method for t(P_fONmax_), t(Pa_max_), P_fONmax_ and Pa_max_ are given in Figure S5.

### Bleach correction of single cell time-series

In time-series of individual cells, bleaching of GFP in these cells occurred. Hence, we applied a bleach correction to our time-series data. After each time-series a new spot was chosen at an appropriate distance to ensure that no bleaching had occurred yet on this spot. Twenty successive images were taken. One image was immediately taken after the previous one. For each cell of this spot the obtained ‘bleach curve’ was fitted exponentially. The resulting rates were averaged. The data obtained in the actual time-series were then divided by e^−nk^, with n being the number of pictures already taken of this spot and k the average of the rates determined by the exponential fit of the ‘bleach curves’.

### GFPmut1 maturation

To determine the time delay between expression of the GFP reporter and the onset of fluorescence, strain TMB 1172 was grown in LB medium as described above. For induction of GFPmut1 expression bacitracin was added after 60 min at a final concentration of 30 µg/ml and erythromycin was added at 80 min, inhibiting protein biosynthesis. Increase of fluorescence after 80 min must therefore be due to folding of already synthesized GFP (Figure S3). Assuming a first-order kinetic we fitted the data with a single exponential function and obtained a characteristic maturation time of 7.9±0.69 min.

## Supporting Information

Table S1 **Fit parameter for the fluorescence distributions given in** **Figure 3** **.**
(DOC)

Table S2 **Fit parameters for the fraction of cells in the ‘ON’ state f_ON_.**
(DOC)

Table S3 **Fit parameter for the switching rate P_fON_.**
(DOC)

Table S4 **Time point of maximal switching rate t(P_fONmax_).**
(DOC)

Table S5 **Maximal switching rate P_fONmax_.**
(DOC)

Table S6 **Fit parameters for the basal fluorescence level FI_basal_.**
(DOC)

Table S7 **Fit parameter for the basal expression rate Pa.**
(DOC)

Table S8 **Maximal basal expression rate Pa_max_.**
(DOC)

Table S9 **Time point of maximal basal expression rate t(Pa_max_).**
(DOC)

Table S10 **Maturation of GFPmut1.**
(DOC)

Figure S1 **Influence of bacitracin on cell growth.** Cells were grown as described in the Material and Methods section in the presence of bacitracin at different final concentrations (Black: 0 µg/ml, grey: 0.1 µg/ml, blue: 0.3 µg/ml, yellow: 1 µg/ml, green: 3 µg/ml, red: 30 µg/ml). At these concentrations bacitracin has no influence on cell growth.
(EPS)

Figure S2 **Expression profiles of the Lia response at 0.1 µg/ml bacitracin.** At these very low inducing concentration of bacitracin nearly all cells stay in the non-induced ‘OFF’ state. A) Representative image of *B. subtilis* cells 60 min after bacitracin induction. B) Histograms of GFP expression from the *liaI* promoter for different time points (T_10_ = 10 min after bacitracin induction). Red arrows indicate the few cells in the ‘ON’ state at this bacitracin concentration.
(EPS)

Figure S3 **Maturation of GFPmut1.** Arrow indicates the addition of 400 µg/ml erythromycin at 80 min leading to immediate translation inhibition. Therefore any fluorescence development arising after erythromycin addition can be attributed to the maturation of the GFP fluorophore. Grey: cells grown in the absence of erythromycin. Black: Cells grown in the presence of erythromycin. Solid lines: best fit to an exponential function (Table S10).
(EPS)

Figure S4 **Flow cytometry analysis of the P** ***_liaI_*** **response of LiaFSR to bacitracin.** Flow cytometry analysis verified the results obtained by quantitative fluorescence microcopy as shown in main Figure 2. Addition of bacitracin induced GFP expression. At T_60_ all cells reached their maximum fluorescence intensities. While at high bacitracin concentrations all cells shifted to high fluorescence values, at low bacitracin concentrations (1 and 0.3 µg/ml) a fraction of cells did not express GFP and stayed at the autofluorescence value. As low fluorescence intensities of induced cells were hard to distinguish from the background fluorescence of not induced cells using flow cytometry, we chose quantitative fluorescence microscopy for detailed analysis of the LiaFSR response. Data shown here represent the mean grey value of each single cell: mean FI [FU]. A) Background signal of the buffer PBS in the gated area. B) Autofluorescence of not induced *Bacillus subtilis* cells C)–F) Histograms of GFP expression from the *liaI* promoter for different time points, at C) 30 µg/ml bacitracin (T_30_ = 30 min after bacitracin induction), D) 3 µg/ml bacitracin, E) 1 µg/ml bacitracin, and F) 0.3 µg/ml bacitracin.
(EPS)

Figure S5 **Maximal switching rate P_fONmax_ and maximal basal expression rate Pa_max_ for various bacitracin concentrations.** As switching into the ‘ON’ state took place in a very short time period of less than 10–15 min, only few data points between the ‘OFF’ and the ‘ON’ state could be obtained. As cells treated with high bacitracin concentrations, although not showing any fitness defects, tended to lyse when stored on ice, a shorter experimental time resolution was not possible. Therefore, to reduce the error by simply fitting to the data, the maximal switching rate as well as the maximal basal expression rate was determined using several calculation methods as described in the Material and Methods section. This Figure gives an overview of the data obtained by the various methods used. A) Time point of maximum switching rate t(P_fONmax_); Black, grey and light grey bars represent data obtained as described in Table S4 a–c. Blue: averaged data of the time point of maximal switching. B) Maximal switching rate P_fONmax_: Black, and light grey bars represent data obtained as described in Table S5 a and b. Blue: averaged data of maximal switching rate P_fONmax_. C) Time point of maximum basal expression rate t(Pa_max_); Black, grey and light grey bars represent data obtained as described in Table S9 a–c. Blue: average data of t(Pa_max_). D) Maximum basal expression rate Pa_max_; Black and light grey bars represent data as described in Table S8 a and b. Blue: average date of maximal Pa_max_.
(EPS)
