# Supplementary material for: Immediate and Heterogeneous Response of the LiaFSR Two-Component System of Bacillus subtilis to the Peptide Antibiotic Bacitracin
Source: PLoS One. 2013 Jan 11;8(1):e53457. doi: 10.1371/journal.pone.0053457 (PMC3543457; doi:10.1371/journal.pone.0053457)
Supplement: Table S1 — Fit parameter for the fluorescence distributions given in Figure 3 . (DOC) [file pone.0053457.s001.doc]

**Table S1: Fit parameter for the fluorescence distributions given in Figure 3.**

| bacitracin  [μg/ml] | width [FU] | X_0_ [FU] | Y_0_ | A |
| --- | --- | --- | --- | --- |
| 30 | 12.2 +- 1.2 | 22.8 +- 0.7 | 0.1 +- 0.2 | 5.7 +- 0.4 |
| 3 | 11.9 +- 0.8 | 32.1 +- 0.5 | 0.1 +- 0.2 | 9.2 +- 0.5 |

Parameter determined from the best fit to a Gaussian function FI(T) = y_0_ + A exp (-((x-x_0_)/width)^2^), with width the width of the Gaussian function, A maximal number, X_0_ point of maximal number, Y_0_ y-value of the Gaussian function at FI_0_.
