# Supplementary material for: Immediate and Heterogeneous Response of the LiaFSR Two-Component System of Bacillus subtilis to the Peptide Antibiotic Bacitracin
Source: PLoS One. 2013 Jan 11;8(1):e53457. doi: 10.1371/journal.pone.0053457 (PMC3543457; doi:10.1371/journal.pone.0053457)
Supplement: Table S2 — Fit parameters for the fraction of cells in the ‘ON’ state fON. (DOC) [file pone.0053457.s002.doc]

**Table S2: Fit parameters for the fraction of cells in the ‘ON’ state f_ON_.**

| bacitracin  [μg/ml] | k [1/min] | T_half_ [min] | f_max_ [%] | f_base_ [%] |
| --- | --- | --- | --- | --- |
| 30 | 0.59 +- 0.1 | 8.3 +- 0.1 | 99 +- 0.2 | 0 +- 0.0 |
| 3 | 2.04 +- 4.5 | 9.7 +- 2.7 | 100 +- 0.0 | 0 +- 0.0 |
| 1 | 1.84 +- 0.3 | 12.7 +- 0.4 | 78 +- 2.4 | 0 +- 0.4 |
| 0.3 | 0.75 +- 0.0 | 13.0 +- 1.1 | 26 +- 1.5 | 0 +- 0.0 |

Parameters determined from the best fit to a sigmoidal function f_ON_(T) = f_base_ + f_max_/ 1+ exp(*k*(T_half_ - T)), with f_base_ baseline, f_max_ maximum fraction of cells in the ‘ON’ state, T_half_ half time and *k* rate.
