# Supplementary material for: Immediate and Heterogeneous Response of the LiaFSR Two-Component System of Bacillus subtilis to the Peptide Antibiotic Bacitracin
Source: PLoS One. 2013 Jan 11;8(1):e53457. doi: 10.1371/journal.pone.0053457 (PMC3543457; doi:10.1371/journal.pone.0053457)
Supplement: Table S3 — Fit parameter for the switching rate PfON. (DOC) [file pone.0053457.s003.doc]

**Table S3: Fit parameter for the switching rate P_fON_.**

| bacitracin  [μg/ml] | width [min] | X_0_ [min] | Y_0_ [%/min] | A [%/min] |
| --- | --- | --- | --- | --- |
| 30 | 4.6 +- 0.2 | 11.6 +- 0.1 | 0.0 +- 0.0 | 12 +- 0.4 |
| 3 | 2.5 +- 1.2 | 12.1 +- 0.4 | 0.0 +- 0.0 | 26 +- 24.2 |
| 1 | 4.6 +- 0.3 | 14.9 +- 0.3 | 0.2 +- 0.2 | 10 +- 0.5 |
| 0.3 | 3.8 +- 0.7 | 15.5 +- 0.8 | 0.0 +- 0.0 | 4 +- 0.6 |

Parameter determined from the best fit to a Gaussian function P_fON_ (T) = y_0_ + Aexp (-((x-x_0_)/width)^2^), with width the width of the Gaussian function, A maximal P_fON_, X_0_ time-point of maximal P_fON_, Y_0_ y-value of the Gaussian function at T_0_.
