# Supplementary material for: Immediate and Heterogeneous Response of the LiaFSR Two-Component System of Bacillus subtilis to the Peptide Antibiotic Bacitracin
Source: PLoS One. 2013 Jan 11;8(1):e53457. doi: 10.1371/journal.pone.0053457 (PMC3543457; doi:10.1371/journal.pone.0053457)
Supplement: Table S4 — Time point of maximal switching rate t(PfONmax). (DOC) [file pone.0053457.s004.doc]

**Table S4: Time point of maximal switching rate t(P_fONmax_).**

| bacitracin  [μg/ml ] | a  [min] | b  [min] | c  [min] | Average  [min] |
| --- | --- | --- | --- | --- |
| 30 | 8.3 ± 0.1 | 14 ± 3.5 | 11.6 ± 0.1 | 11.3 ± 1.3 |
| 3 | 9.7 ± 2.7 | 10 ± 2.5 | 12.1 ± 0.4 | 10.6 ± 1.9 |
| 1 | 12.7 ± 0.4 | 15 ± 2.5 | 14.9 ± 0.3 | 14.2 ± 1.1 |
| 0.3 | 13.0 ± 1.1 | 15 ± 2.5 | 15.5 ± 0.8 | 14.3 ± 1.5 |

The time point of the maximal switching rate has been determined in three different ways: a) T_half_ of the sigmoidal fit applied to Figure 4 left according to f_ON_(T) = f_base_ + f_max_/ 1+ exp(k(T_half_ – T)). b) Time point of P_fONmax_ = maximum of the 1^st^ derivative of the exact data points of f_ON_. c) by obtaining x_0_ of the Gaussian fit applied to the data in Figure 4 right according to P_fON_(T) = y_0_ + A exp (-((x-x_0_)/width)^2^).
