# Supplementary material for: Immediate and Heterogeneous Response of the LiaFSR Two-Component System of Bacillus subtilis to the Peptide Antibiotic Bacitracin
Source: PLoS One. 2013 Jan 11;8(1):e53457. doi: 10.1371/journal.pone.0053457 (PMC3543457; doi:10.1371/journal.pone.0053457)
Supplement: Table S5 — Maximal switching rate PfONmax. (DOC) [file pone.0053457.s005.doc]

**Table S5: Maximal switching rate P_fONmax_.**

| bacitracin  [μg/ml] | A  [%/min] | b  [%/min] | Average  [%/min] |
| --- | --- | --- | --- |
| 30 | 9 ± 1.7 | 12 ± 0.4 | 10.6 ± 1.1 |
| 3 | 13 ± 3.4 | 26 ± 24.2 | 19.5 ± 13.8 |
| 1 | 9.3 ± 5.2 | 10 ± 0.5 | 9.7 ± 2.9 |
| 0.3 | 3.7 ± 4.5 | 4 ± 0.6 | 3.9 ± 2.6 |

The maximal switching rate has been determined in two different ways: a) P_fonmax_ = maximum of the 1^st^ derivative of the exact data points of f_ON_. b) by obtaining A of the Gaussian fit applied to the data Figure 4 right according to P_fON_(T) = y_0_ + Aexp (-((x-x_0_)/width)^2^). The high error for data determined at 3 μg/ml bacitracin is attributed to the steep increase of the fraction of cells in the ‘ON’ state leading to a high fitting error. Additional data points in order to reduce the error could not be attained, as cells stored on ice for later image acquisition tended to lyse at bacitracin concentrations > 1 μg/ml. Therefore image acquisition was performed immediately after sampling of the cells.
