# Supplementary material for: Immediate and Heterogeneous Response of the LiaFSR Two-Component System of Bacillus subtilis to the Peptide Antibiotic Bacitracin
Source: PLoS One. 2013 Jan 11;8(1):e53457. doi: 10.1371/journal.pone.0053457 (PMC3543457; doi:10.1371/journal.pone.0053457)
Supplement: Table S6 — Fit parameters for the basal fluorescence level FIbasal. (DOC) [file pone.0053457.s006.doc]

**Table S6: Fit parameters for the basal fluorescence level FI_basal_.**

| bacitracin  [μg/ml] | k [1/min] | T_half_ [min] | f_max_ [FU] | f_base_ [FU] |
| --- | --- | --- | --- | --- |
| 1 | 0.26 +- 0.1 | 5.5 +- 0.5 | 21.6 +- 1.1 | 10.5 +- 0.5 |
| 0.3 | 0.45 +- 0.6 | 5.5 +- 0.6 | 11.9 +- 0.4 | 8.5 +- 0.2 |

Parameters determined from the best fit to a sigmoidal function FI(T) = f_base_ + f_max_/ 1+ exp(*k*(T_half_ – T)), with f_base_ baseline, f_max_ maximum basal fluorescence intensity, T_half_ half time and *k* rate.
