# Supplementary material for: Immediate and Heterogeneous Response of the LiaFSR Two-Component System of Bacillus subtilis to the Peptide Antibiotic Bacitracin
Source: PLoS One. 2013 Jan 11;8(1):e53457. doi: 10.1371/journal.pone.0053457 (PMC3543457; doi:10.1371/journal.pone.0053457)
Supplement: Table S7 — Fit parameter for the basal expression rate Pa. (DOC) [file pone.0053457.s007.doc]

**Table S7: Fit parameter for the basal expression rate Pa.**

| bacitracin  [μg/ml] | width [min] | X_0_ [min] | Y_0_ [FU/min] | A [FU/min] |
| --- | --- | --- | --- | --- |
| 1 | 2.5 +- 0.2 | 8.6 +- 0.1 | 0.0 +- 0.1 | 2.7 +- 0.2 |
| 0.3 | 6.5 +- 1.7 | 8.1 +- 0.8 | 0.0 +- 0.1 | 0.3 +- 0.1 |

Parameter determined from the best fit to a Gaussian function Pa(T) = y_0_ + A exp (-((x-x_0_)/width)^2^), with width the width of the Gaussian function, A maximal Pa, X_0_ time-point of maximal Pa, Y_0_ y-value of the Gaussian function at T_0_.
