# Supplementary material for: Immediate and Heterogeneous Response of the LiaFSR Two-Component System of Bacillus subtilis to the Peptide Antibiotic Bacitracin
Source: PLoS One. 2013 Jan 11;8(1):e53457. doi: 10.1371/journal.pone.0053457 (PMC3543457; doi:10.1371/journal.pone.0053457)
Supplement: Table S8 — Maximal basal expression rate Pamax. (DOC) [file pone.0053457.s008.doc]

**Table S8: Maximal basal expression rate Pa_max_.**

| bacitracin  [μg/ml] | A  [FU/min] | b  [FU/min] | Average  [FU/min] |
| --- | --- | --- | --- |
| 1 | 2 ± 0.5 | 2.7 ± 0.2 | 2.3 ± 0.4 |
| 0.3 | 0.3 ± 0.4 | 0.3 ± 0.1 | 0.3 ± 0.3 |

The maximal basal expression rate has been determined in two different ways: a) Pa = maximum of the 1^st^ derivative of the exact data points of FI. b) by obtaining x_0_ of the Gaussian fit applied to Figure 5 C, D according to Pa(T) = y_0_ + Aexp (-((x-x_0_)/width)^2^).
