# Supplementary material for: Immediate and Heterogeneous Response of the LiaFSR Two-Component System of Bacillus subtilis to the Peptide Antibiotic Bacitracin
Source: PLoS One. 2013 Jan 11;8(1):e53457. doi: 10.1371/journal.pone.0053457 (PMC3543457; doi:10.1371/journal.pone.0053457)
Supplement: Table S9 — Time point of maximal basal expression rate t(Pamax). (DOC) [file pone.0053457.s009.doc]

**Table S9: Time point of maximal basal expression rate t(Pa_max_).**

| bacitracin  [μg/ml] | a  [min] | b  [min] | c  [min] | Average  [min] |
| --- | --- | --- | --- | --- |
| 1 | 5.5 ± 0.5 | 10 ± 2.5 | 8.6 ± 0.7 | 8.0 ± 0.4 |
| 0.3 | 5.5 ± 0.6 | 5 ± 2.5 | 8.1 ± 0.8 | 6.2 ± 1.3 |

The time point of the maximal basal expression rate has been determined in three different ways: a) T_half_ of the sigmoidal fit applied to Figure 5 A, C according to FI(T) = f_base_ + f_max_/ 1+ exp(k(T_half_ - T)). b) Pa = maximum of the 1^st^ derivative of the exact data points of FI. c) by obtaining x_0_ of the Gaussian fit applied to Figure 5 right according to Pa(T) = y_0_ + Aexp (-((x-x_0_)/width)^2^).
