# Supplementary material for: Immediate and Heterogeneous Response of the LiaFSR Two-Component System of Bacillus subtilis to the Peptide Antibiotic Bacitracin
Source: PLoS One. 2013 Jan 11;8(1):e53457. doi: 10.1371/journal.pone.0053457 (PMC3543457; doi:10.1371/journal.pone.0053457)
Supplement: Table S10 — Maturation of GFPmut1. (DOC) [file pone.0053457.s010.doc]

**Table S10: Maturation of GFPmut1.**

| Fit number | Y_0_  [FU] | A  [FU] | invTau  [1/min] | MT  [min] |
| --- | --- | --- | --- | --- |
| 1 | 1.04 ± 0.02 | - 1141 ± 0.01 | 0.12 ± 0.04 | 8.33 |
| 2 | 1.04 ± 0.01 | - 579 ± 0.1 | 0.12 ± 0.01 | 8.33 |
| 3 | 1.03 ± 0.01 | - 6351 ± 0.1 | 0.14 ± 0.01 | 7.14 |

Given are the parameter of the exponential fit y = y_0_ + Aexp (- invTau · x) applied to data in Figure S3. The maturation time (MT [min]) is the invers of invTau.
